# Sodium-Glucose Cotransporter 2 (SGLT2) Inhibitors from Natural Products: Discovery of Next-Generation Antihyperglycemic Agents

**DOI:** 10.3390/molecules21091136

**Published:** 2016-08-27

**Authors:** Chang-Ik Choi

**Affiliations:** College of Pharmacy, Dongguk University-Seoul, Goyang 10326, Korea; cichoi@dongguk.edu; Tel.: +82-31-961-5230

**Keywords:** type 2 diabetes mellitus, sodium-glucose cotransporter, SGLT2 inhibitor, natural product, phlorizin

## Abstract

Diabetes mellitus is a chronic condition associated with the metabolic impairment of insulin actions, leading to the development of life-threatening complications. Although many kinds of oral antihyperglycemic agents with different therapeutic mechanisms have been marketed, their undesirable adverse effects, such as hypoglycemia, weight gain, and hepato-renal toxicity, have increased demand for the discovery of novel, safer antidiabetic drugs. Since the important roles of the sodium-glucose cotransporter 2 (SGLT2) for glucose homeostasis in the kidney were recently elucidated, pharmacological inhibition of SGLT2 has been considered a promising therapeutic target for the treatment of type 2 diabetes. Since the discovery of the first natural SGLT2 inhibitor, phlorizin, several synthetic glucoside analogs have been developed and introduced into the market. Furthermore, many efforts to find new active constituents with SGLT2 inhibition from natural products are still ongoing. This review introduces the history of research on the development of early-generation SGLT2 inhibitors, and recent progress on the discovery of novel candidates for SGLT2 inhibitor from several natural products that are widely used in traditional herbal medicine.

## 1. Introduction

Diabetes mellitus is a chronic condition characterized by hyperglycemia, due to the metabolic impairment of insulin production/secretion and/or utilization in human body. It plays a pivotal role in the development of several important cardiovascular, neurological, and renal complications, which are the main underlying reasons for morbidity and mortality in diabetic patients [1]. The International Diabetes Federation recently reported that the estimated number of adult people with diabetes worldwide is approximately 415 million, indicating that one in eleven adults currently has diabetes, and this number is expected to increase to over 640 million within 25 years [2].

Type 2 diabetes mellitus, also called non-insulin dependent diabetes mellitus (NIDDM), is the most common type of diabetes and its pathogenesis is associated with various mechanisms. Due to the complexity of this disease and the maintenance of compliance of the diabetic patients, many kinds of oral antihyperglycemic agents have been developed and are now available, such as sulfonylureas, meglitinides, biguanides, thiazolidinediones, α-glucosidase inhibitors, and dipeptidyl peptidase-IV (DPP-IV) inhibitors. Nevertheless, some undesirable adverse effects caused by the administration of these medications, including hypoglycemia, gastrointestinal symptoms, weight gain, and hepato-renal toxicity, have increased demand for the discovery of safer antidiabetic agents with novel therapeutic mechanisms [3].

After the renal glucose reabsorption kinetics were firstly demonstrated in the late 1930s [4], the two main glucose transport systems in the proximal tubule were characterized as the sodium-glucose co-transporter (SGLT) 1 (high affinity, low capacity) and SGLT2 (low affinity, high capacity) [5,6,7,8,9]. SGLT2, the most prevalent and important SGLT subtype, accounts for more than 90% of glucose reabsorption at the early proximal tubule in normoglycemic conditions [10,11,12]. On the other hand, hyperglycemia stimulates proximal tubular growth and SGLT2 expression, leading to an increase in renal glucose reabsorption and the unsatisfactory control of diabetes. The inhibition of SGLT2 induces glucosuria and lowers blood glucose levels [12,13,14,15,16]. As a key mechanism for glucose homeostasis in the kidney, therefore, SGLT2 inhibitors have been considered promising agents for the treatment of type 2 diabetes in the past few years.

Natural products have been widely used for the regulation of normal physiological conditions and the treatment of various chronic diseases, as a form of pharmaceuticals and dietary supplements. To date, numerous studies have demonstrated that natural product extracts and/or their active phytochemicals showed various antidiabetic properties, such as insulinotropic effect, peroxisome proliferator-activated receptor γ (PPARγ) activation, AMP-activated protein kinase (AMPK) pathway activation, α-glucosidase inhibition, glucose transporter 4 (GLUT4) expression/translocation, and protein tyrosine phosphatase 1B (PTP1B) inhibition, with lower side effects [3]. This review introduces the first plant-derived SGLT inhibitory compound, phlorizin, and its synthetic analogs, which are now commercially available, and summarizes recent progress on the discovery of novel candidates for SGLT2 inhibitor from natural products.

## 2. The First Natural SGLT Inhibitor, Phlorizin, and Its Glucoside Analogs

Phlorizin, a dihydrochalcone isolated from the bark of apple trees in 1835, is known to be the first natural product substance with SGLT inhibitory activity [17]. Due to its similarities with extracts from the cinchona and willow tree, phlorizin was previously considered a candidate for the treatment of fevers, infectious diseases, and malaria [18]. Approximately 50 years later, Chasis et al. [19] reported that phlorizin inhibits renal glucose reabsorption and increases urinary glucose excretion. The association between phlorizin and the active glucose transport system of the proximal tubule brush border was revealed in the early 1970s [5]. Moreover, several in vivo studies using diabetic animal models have demonstrated that phlorizin administration decreased fasting and/or postprandial blood glucose levels and increased insulin sensitivity [20,21,22,23]. More recently, Katsuno et al. [24] reported that phlorizin inhibited both human SGLT1 and SGLT2, with the inhibitory constant (*Ki*) values of 151 and 18.6 nM, respectively.

In spite of its sufficient inhibitory effects against SGLTs, phlorizin was ultimately considered to be inappropriate for further development as an antihyperglycemic medication due to some critical drawbacks. First, as described above, phlorizin inhibits both SGLT1 and SGLT2 with low therapeutic selectivity. The inhibition of SGLT1, which is primarily localized in the small intestine, can cause several gastrointestinal side effects, such as diarrhea, dehydration, and malabsorption. Second, phlorizin is poorly absorbed in the small intestine, due to its low oral bioavailability. Third, phloretin, a β-glycosidases catalyzed hydrolytic metabolite of phlorizin, strongly inhibits the ubiquitous glucose transporter 1 (GLUT1), which then may obstruct glucose uptake in various tissues [9,17,24,25].

To overcome these limitations, several pharmaceutical companies began extensive research to develop novel phlorizin-based analogs with improved bioavailability and stability, as well as SGLT2 selectivity. In the early stages, they focused on the *O*-glucoside analogs, and an orally-available selective SGLT2 inhibitor T-1095 was developed [26]. T-1095 underwent extensive hepatic metabolism into its active form, T-1095A, resulting in a dose-dependent decrease in urinary glucose reabsorption and suppression of blood glucose elevation, along with increased urinary glucose excretion. The calculated IC_50_ values of T-1095A for the human SGLT1 and SGLT2 were approximately 200 nM and 50 nM, respectively, which reflected more selective and potent inhibitory activities when compared with phlorizin [26]. Other *O*-glucoside derivatives sergliflozin [27], remogliflozin [28], and AVE2268 [29,30] were developed and their pharmacokinetic and/or pharmacodynamics properties have been evaluated in various in vivo and clinical settings [31,32,33,34,35,36,37,38,39,40,41]. Although these *O*-glucoside inhibitors showed minimized glucosidase-mediated degradation and enhanced systemic exposure, their poor pharmacokinetic stability and incomplete pharmacological selectivity for SGLT2 turned the interest of many scientists and pharmaceutical companies toward the another derivatives of phlorizin, *C*-glucosides.

**Figure 1:**
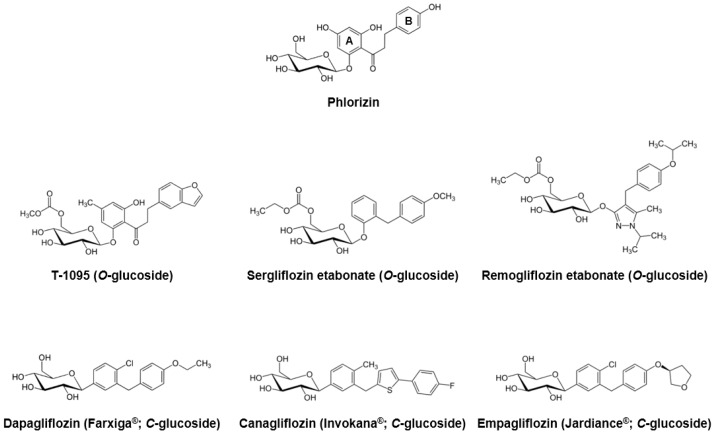
Phlorizin and its major *O*- and *C*-glucoside analogs.

Since the first synthesis of phlorizin *C*-glucoside analogs was performed in 2000 [42], there have been numerous attempts to find optimal substituents with sufficient potency and selectivity against SGLT2. Consequently, in 2008, Meng et al. [43] developed dapagliflozin, with lipophilic ethoxy substituents at the 4-position on the B-ring of phlorizin (Figure 1). Dapagliflozin showed more than 1200-fold higher potency for human SGLT2 [IC_50_ (nM): 1.12] versus SGLT1 [IC_50_ (nM): 1391]. Dose-dependent glucosuric response and decreased fasting and postprandial blood glucose levels were also observed after oral administration of dapagliflozin to rats [43,44]. As an innovative antidiabetic candidate, the efficacy of dapagliflozin has been evaluated in many clinical studies, and this agent exhibited significant decreased plasma glucose levels and glycated hemoglobin (HbA1c), improved glycemic control, and body weight reduction [45,46,47,48,49,50,51,52]. Dapagliflozin was first approved and marketed in Europe in 2012, and the United States Food and Drug Administration (FDA) committee also approved this medication for the treatment of type 2 diabetes in January, 2014.

Beginning with the appearance of dapagliflozin, several *C*-glucoside inhibitors have been subsequently developed. Canagliflozin, characterized by a thiophene derivative of *C*-glucoside [53], was approved by the FDA in 2013. Along with over 400-fold difference in inhibitory activities between human SGLT1 and SGLT2 [IC_50_ (nM) SGLT1: 910; SGLT2: 2.2] [53], canagliflozin exhibited good antihyperglycemic properties comparable to dapagliflozin in many clinical practices [54,55,56,57]. Empagliflozin was the third agent in the gliflozin class approved by both the European Medicines Agency (EMA) and FDA in 2014, having the highest selectivity for SGLT2 over SGLT1 (approximately 2700-fold) among the SGLT2 inhibitors on the market [58]. In recent years, many Japanese pharmaceutical industries have led the development of next generation SGLT2 inhibitors, including ipragliflozin, tofogliflozin, and luseogliflozin [25]. Other compounds, ertugliflozin and LX-4211 (sotagliflozin, a dual SGLT1/2 inhibitor), are now in late-phase clinical trials [59].

## 3. Recent Progress on the Discovery of Novel SGLT2 Inhibitors from Natural Products

**Figure 2:**
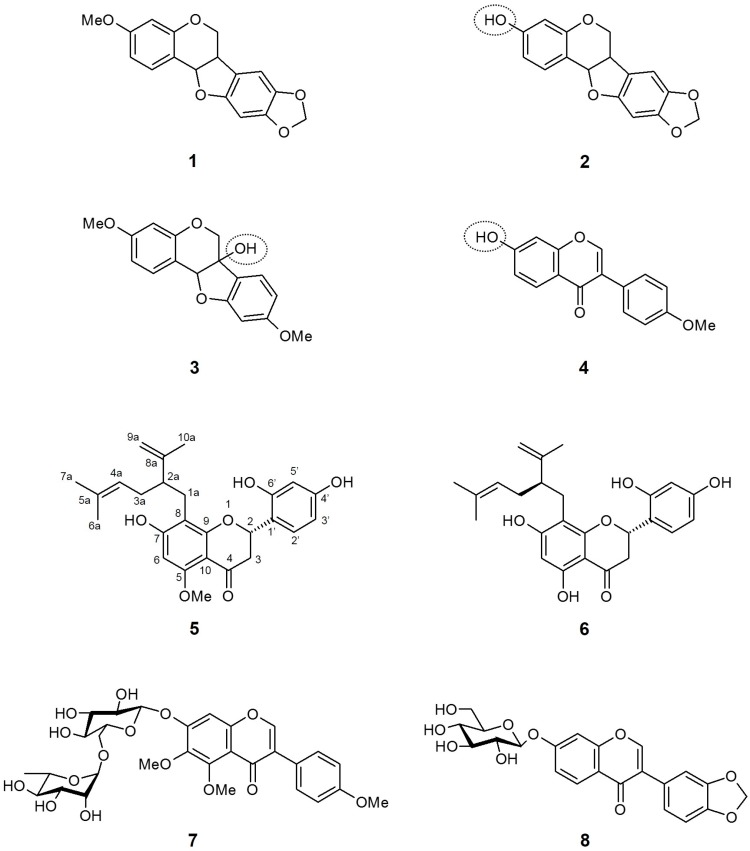
Chemical structures of active compounds from *Sophora flavescens* [60,61].

**Figure 3:**
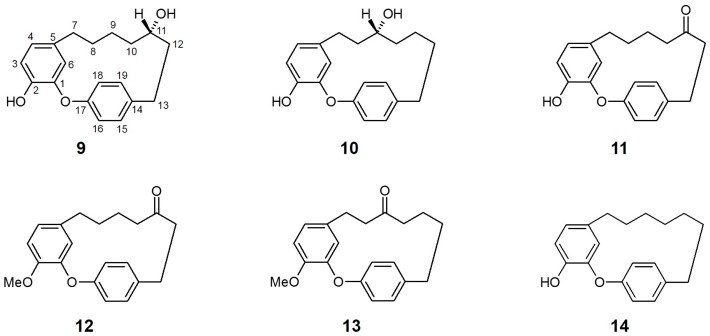
Chemical structures of active compounds from *Acer nikoense* [62].

**Figure 4:**
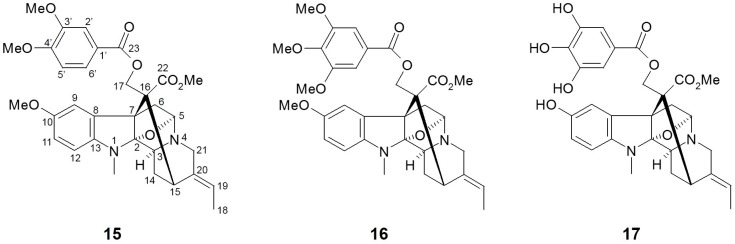
Chemical structures of active compounds from *Alstonia macrophylla* [63].

**Figure 5:**
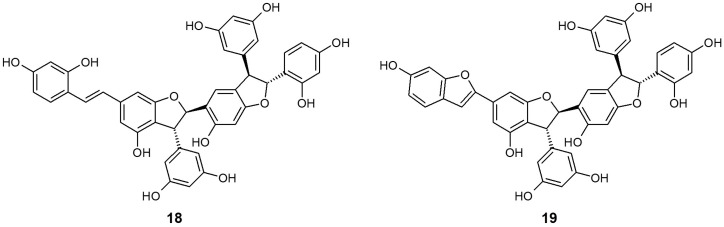
Chemical structures of active compounds from *Gnetum gnemonoides* [64].

Although many SGLT2 inhibitors are now available and extensively used, many efforts to find new active compounds from natural products as viable candidates for novel SGLT2 inhibitor, like phlorizin, are still ongoing. The chemical structures and assay results of several major natural compounds are described in Figure 2, Figure 3, Figure 4 and Figure 5 and Table 1.

### 3.1. Sophora flavescens (Fabaceae)

*Sophora flavescens* (*S. flavescens*) is one of the most popular and important traditional Chinese medicines. The root of this species (known as “Kushen”) is widely used for the treatment of many diseases, including fever, dysentery, jaundice, leukorrhea, pyogenic skin infection, scabies, swelling, and pain. To date, over 200 constituents have been isolated from *S. flavescens*, and the major component of this plant were alkaloids and flavonoids [65]. In addition to its traditional uses, numerous studies have been performed to find other therapeutic effects of *S. flavescens*, such as antitumor, anti-inflammatory, anti-nociceptive, anti-anaphylaxis, anti-asthma, antimicrobial, cardiovascular-protective, and immunoregulatory effects, using its crude extracts and major active compounds [65].

Sato et al. [60], as a follow-up research from the discovery that the *S. flavescens* methanol extract has a potent SGLT inhibitory activity, screened nine compounds isolated from the dried root of *S. flavescens* for their effects on SGLT inhibition. Interestingly, except for pterocarpin (**1**), three compounds with isoflavonoid-based structures, namely maackiain (**2**), variabilin (**3**), and formononetin (**4**) showed exclusive inhibitory activity only against SGLT2, but not against SGLT1. Thus, it is suggested that the presence of the hydroxyl functional group in isoflavonoid is crucial for acquiring SGLT2 inhibitory activity. Meanwhile, most flavanone compounds extensively inhibited both SGLTs, with selectivity against SGLT2. The two most potent compounds were (−)–kurarinone (**5**) and sophoraflavanone G (**6**), with IC_50_ values of 10.4 and 18.7 μM for SGLT1, and 1.7 and 4.1 μM for SGLT2, respectively. The increase in SGLT1 inhibition was assumed to be attributable to the common lavandulyl functional group at the *C*-8 position.

More recently, Yang et al. [61] reported the effects of isoflavonoid glycosides from the root of *S. flavescens* on SGLT2 inhibition. All nine isolated compounds in the research exhibited SGLT2 inhibitory activity, with the strongest SGLT2 inhibition in compound **7** [IC_50_ (μM): 2.6 ± 0.18]. In addition, compound **8** also showed moderate inhibition for SGLT2 [IC_50_ (μM): 15.3 ± 1.44]. Since the study was designed as a simple screening assessment and the inhibitory activity was established only for SGLT2, the conformational importance of the inhibitory activity and selectivity against SGLT2 was not discussed.

### 3.2. Acer nikoense (Aceraceae)

*Acer nikoense* (*A. nikoense*) is native to and widely distributed in Japan, and its stem bark extracts are used in Japanese folk medicine for the treatment of hepatic disorders and eye diseases [66]. Several active constituents from *A. nikoense*, including specific cyclic diarylheptanoid acerogenins, have been assessed for their anticancer, anti-inflammatory, antifungal, and antibacterial effects [62].

Morita et al. [64] evaluated the effects of four compounds isolated from the bark of *A. nikoense* and sixteen related derivatives on SGLT inhibitory activity. Two cyclic diarylheptanoids, acerogenin A (**9**) and B (**10**), presented marked inhibition for both SGLT1 [IC_50_ (μM) **9**: 20.0; **10**: 26.0] and SGLT2 [IC_50_ (μM) **9**: 94.0; **10**: 43.0], while other isolated compounds (including one acyclic diarylheptanoid) did not show sufficient inhibition of either SGLT.

Notably, the ketone derivatives at the *C*-11 position (with or without additional substitution of the hydroxyl group for methyl ester at *C*-2) of acerogenin A/B, compounds **11** (acerogenin C), **12**, and **13** acquired increased, selective inhibitory activity against SGLT2 versus SGLT1 compared to their precursors at the same tested concentration. Moreover, another derivative of acerogenin A, characterized by dihydroxylation at the *C*-11 position (**14**) showed the most potent inhibitory activities against both SGLTs, with non-selectivity. These results suggest that the position and/or presence of a hydroxyl group at *C*-9 or *C*-11 and their stereochemistry may not be a key structure for SGLT inhibition. The modification of the diarylheptanoid ring system (such as amide substitution or unsaturated C–C bond formation) resulted in a decrease in SGLT inhibition, indicating that the ring conformation of acerogenins and its derivatives can affect the inhibitory effect of each constituent against SGLTs.

### 3.3. Alstonia macrophylla (Apocynaceae)

*Alstonia macrophylla* (*A. macrophylla*), also called hard alstonia or hard milkwood, is native to Southeast Asia regions such as Indonesia, Malaysia, the Philippines, Thailand, and Vietnam. This plant has been traditionally used as a general tonic, aphrodisiac, anticholeric, antidysenteric, antipyretic, emmenagogue, and vulnerary agent in Thailand [67]. As a rich source of diverse phytochemicals, several biological activities including antimalarial, antimicrobial, antioxidant, antidiabetic, anti-inflammatory, antipyretic, antipsychotic, antifertility, and antiprotozoal effects have been demonstrated using various extracts and active compounds from *A. macrophylla* [68].

Arai et al. [63] isolated twenty alkaloid compounds from the leaves of *A. macrophylla* and assessed the SGLT inhibitory potentials of these constituents. Of twenty compounds, five picraline-type alkaloids showed good SGLT1 and SGLT2 inhibition, with the highest in 10-methoxy-*N*(1)-methylburnamine-17-*O*-veratrate [**15**, IC_50_ (μM) SGLT1: 4.0; SGLT2: 0.5] and alstiphyllanine D [**16**, IC_50_ (μM) SGLT1: 5.0; SGLT2: 2.0]. Meanwhile, other ajmaline- and macroline-type alkaloid compounds showed no inhibitory activities against SGLT1 and/or SGLT2. The results from the structure-activity relationship (SAR) approach suggested that the presence of an ester side chain at the *C*-17 position may play a pivotal role in SGLT inhibitory activity. When further SAR study using eight derivatives was assessed, the hydroxyl derivative in alstiphyllanine D (**17**) improved the selectivity for SGLT2 more than the others, although the absolute IC_50_ value was slightly increased [IC_50_ (μM) SGLT1: 50.0; SGLT2: 7.0].

### 3.4. Gnetum gnemonoides (Gnetaceae)

*Gnetum gnemonoides* (*G. gnemonoides*) is a kind of tropical lianas, widely distributed in the Southeast Asia-Pacific region including Malaysia, Indonesia, Philippines, New Guinea, and Bismarck Archipelago. Although there is no scientific report on the medicinal effectiveness of *G. gnemonoides* yet, it is known that stilbenes isolated from the *Gnetum* species possess biological properties such as hepatoprotective, antioxidant, antimicrobial, and enzyme inhibitory activities [68].

Shimokawa et al. [64] isolated two stilbene trimers, gneyulin A (**18**) and B (**19**), consisting of oxyresveratrol units from the dried bark of *G. gnemonoides*, and simply screened their effect on the inhibition of SGLT1 and SGLT2. These two compounds both showed moderate, non-selective inhibitory activity for each SGLT [IC_50_ (μM) **18** SGLT1: 27.0, SGLT2: 25.0; **19** SGLT1: 37.0, SGLT2: 18.0]. They also newly discovered two dihydroflavonol-*C*-glucosides, noidesol A and B, but these compounds showed no SGLT inhibitory potential.

### 3.5. Schisandra chinensis (Schisandraceae)

*Schisandra chinensis* (*S. chinensis*, commonly called “five-flavor berry”) is native to Northern China and the Russian Far East, and its fruits are traditionally used as an anti-aging, antitussive, sedative, and tonic agent [69]. *S. chinensis* contains various phytochemicals including polyphenols, lignans, and triterpenoids, and its pharmacological effects on various organ systems have been extensively assessed [70].

Qu et al. [71] recently evaluated the SGLT inhibitory activities of Schisandrae Chinensis Fructus (SCF), for the purpose of identifying specific SGLT2 inhibitory compounds. In the first-step screening with aqueous and ethanol SCF extracts at a concentration with no cytotoxicity (1 mg/mL), more potent inhibition rates for both SGLTs were observed with the ethanol extract. After fractionation of the ethanol extract of SCF, a total of nine fractions (F1–F9) underwent further SGLT inhibition assessment. Of six fractions that exhibited inhibitory activity against SGLT1 and/or SGLT2, only two fractions (F8 and F9) showed significant SGLT2-selective patterns, with the inhibition rate of 41.9% and 36.7% of the control, respectively.

The effects of three common lignan compounds isolated from F8, deoxyschisandrin, schisandrin B (γ-schisandrin), and schisandrin on SGLT inhibition were finally investigated. However, none of them showed inhibitory activities against either SGLT, suggesting that these major lignans are not the primary components for the SGLT inhibition of SCF.

## 4. Conclusions 

Since the important roles of SGLT2 for renal glucose reabsorption and systemic glucose homeostasis in the human body were elucidated, the inhibition of SGLT2 has been considered a promising therapeutic target for the treatment of type 2 diabetes mellitus. From the beginning of the 21st century, several SGLT2 inhibitors derived from a natural active compound phlorizin, began to be marketed and widely used as monotherapy or in combination with other oral antihyperglycemic agents. Although the therapeutic efficacy of these early-generation SGLT2 inhibitors are well established and most of the reported adverse events associated with drug administration tend to be mild and reversible, we should also not rule out the possibility of unrecognized, unexpected side effects which may be fatal and life-threatening to diabetic patients. It is reported that additional adverse reactions, such as ketoacidosis, urosepsis, pyelonephritis, acute kidney injury, anaphylaxis, and angioedema, have been identified as an early post-marketing experience of canagliflozin and empagliflozin use [72,73]. Furthermore, conventional SGLT2 inhibitors have some pharmacokinetic concerns, such as low tissue permeability, poor stability [74], and potential for drug interactions, closely related to their glycoside structure. In a recent clinical study, for example, co-administration of canagliflozin with rifampin, known as a non-selective UDP-glucuronosyltransferase (UGT) enzyme inducer, reduced UGT-mediated metabolism of canagliflozin, leading to a decrease in the plasma exposure of this agent [75].

In this review, various active compounds from several natural products with different chemical and biological characteristics from phlorizin and its synthetic analogs were summarized. Although the recent researches on discovering novel SGLT2 inhibitors from natural products are in its beginning stage and the study results are limited to the in vitro screening of SGLT inhibitory activity and SAR approach, further investigations will provide better molecular information on the discovery and development of next-generation SGLT2 inhibitors which are more effective and safer than conventional drugs.

## Figures and Tables

**Table 1:** SGLT inhibitory properties of several active compounds isolated from natural products.

No.	Species (Family)	Compound Name (If Known)	Inhibition % ^1^		IC_50_ (μM)		Ref.
			SGLT1	SGLT2	SGLT1	SGLT2	
1	*Sophora flavescens* (Fabaceae)	Pterocarpin	NI ^2^	NI ^2^			[60]
2		Maackiain	NI ^2^	66.5			
3		Variabilin	NI ^2^	49.9			
4		Formononetin	NI ^2^	75.4			
5		(−)−Kurarinone	98.8	99.7	10.4	1.7	
6		Sophoraflavanone G	99.2	100.0	18.7	4.1	
7	*Sophora flavescens* (Fabaceae)					2.6 ± 0.18	[61]
8						15.3 ± 1.44	
9	*Acer nikoense* (Aceraceae)	Acerogenin A	92.7	33.9	20.0	94.0	[62]
10		Acerogenin B	94.2	54.2	26.0	43.0	
11		Acerogenin C	44.9	67.8			
12			44.6	53.3			
13			37.7	65.4			
14			96.3	77.4			
15	*Alstonia macrophylla* (Apocynaceae)	10-Methoxy-*N*(1)-methylburnamine-17-*O*-veratrate	95.8	102.6	4.0	0.5	[63]
16		Alstiphyllanine D	89.9	101.4	5.0	2.0	
17			46.9	95.6	20.0	7.0	
18	*Gnetum gnemonoides* (Gnetaceae)	Gneyulin A			27.0	25.0	[64]
19		Gneyulin B			37.0	18.0	

^1^ Inhibition % at 50 μM; ^2^ no or less than 25% inhibition.

## References

[1] Baynes J.W. (1991). Role of oxidative stress in development of complications in diabetes. Diabetes.

[2] International Diabetes Federation (2015). IDF Diabetes Atlas.

[3] Hung H.Y., Qian K., Norris-Natschke S.L., Hsu C.S., Lee K.H. (2012). Recent discovery of plant-derived anti-diabetic natural products. Nat. Prod. Rep..

[4] Shannon J.A., Fisher S. (1938). The renal tubular reabsorption of glucose in the normal dog. Am. J. physiol..

[5] Vick H., Diedrich D.F., Baumann K. (1973). Reevaluation of renal tubular glucose transport inhibition by phlorizin analogs. Am. J. Physiol..

[6] Turner R.J., Moran A. (1982). Heterogeneity of sodium-dependent d-glucose transport sites along the proximal tubule: Evidence from vesicle studies. Am. J. Physiol..

[7] Hediger M.A., Coady M.J., Ikeda T.S., Wright E.M. (1987). Expression cloning and cDNA sequencing of the Na^+^/glucose co-transporter. Nature.

[8] Kanai Y., Lee W.S., You G., Brown D., Hediger M.A. (1994). The human kidney low affinity Na^+^/glucose cotransporter SGLT2. Delineation of the major renal reabsorptive mechanism for D-glucose. J. Clin. Investig..

[9] Wright E.M., Loo D.D., Hirayama B.A. (2011). Biology of human sodium glucose transporters. Physiol. Rev..

[10] Vallon V., Platt K.A., Cunard R., Schroth J., Whaley J., Thomson S.C., Koepsell H., Rieg T. (2011). SGLT2 mediates glucose reabsorption in the early proximal tubule. J. Am. Soc. Nephrol..

[11] Gorboulev V., Schürmann A., Vallon V., Kipp H., Jaschke A., Klessen D., Friedrich A., Scherneck S., Rieg T., Cunard R. (2012). Na^+^-d-glucose cotransporter SGLT1 is pivotal for intestinal glucose absorption and glucose-dependent incretin secretion. Diabetes.

[12] Rieg T., Masuda T., Gerasimova M., Mayoux E., Platt K., Powell D.R., Thomson S.C., Koepsell H., Vallon V. (2014). Increase in SGLT1-mediated transport explains renal glucose reabsorption during genetic and pharmacological SGLT2 inhibition in euglycemia. Am. J. Physiol. Renal Physiol..

[13] Farber S.J., Berger E.Y., Eaerle D.P. (1951). Effect of diabetes and insulin on the maximum capacity of the renal tubules to reabsorb glucose. J. Clin. Investig..

[14] Vestri S., Okamoto M.M., de Freitas H.S., Aparecida Dos Santos R., Nunes M.T., Morimatsu M., Heimann J.C., Machado U.F. (2001). Changes in sodium or glucose filtration rate modulate expression of glucose transporters in renal proximal tubular cells of rat. J. Membr. Biol..

[15] Rahmoune H., Thompson P.W., Ward J.M., Smith C.D., Hong G., Brown J. (2005). Glucose transporters in human renal proximal tubular cells isolated from the urine of patients with non-insulin-dependent diabetes. Diabetes.

[16] Vallon V. (2015). The mechanisms and therapeutic potential of SGLT2 inhibitors in diabetes mellitus. Annu. Rev. Med..

[17] Ehrenkranz R.R.L., Lewis N.G., Kahn C.R., Roth J. (2005). Phlorizin: A review. Diabetes Metab. Res. Rev..

[18] White J.R. (2010). Apple trees to sodium glucose co-transporter inhibitors: A review of SGLT2 inhibition. Clin. Diabetes.

[19] Chasis H., Jolliffe N., Smith H.W. (1933). The action of phlorizin on the excretion of glucose, xylose, sucrose, creatinine and urea by man. J. Clin. Investig..

[20] Rossetti L., Smith D., Shulman G.I., Papachristou D., DeFronzo R.A. (1987). Correction of hyperglycemia with phlorizin normalizes tissue sensitivity to insulin in diabetic rats. J. Clin. Investig..

[21] Dimitrakoudis D., Vranic M., Klip A. (1992). Effects of hyperglycemia on glucose transporters of the muscle: Use of the renal glucose reabsorption inhibitor phlorizin to control glycemia. J. Am. Soc. Nephrol..

[22] Jonas J.C., Sharma A., Hasenkamp W., Ilkova H., Patane G., Laybutt R., Bonner-Weir S., Weir G.C. (1999). Chronic hyperglycemia triggers loss of pancreatic β cell differentiation in an animal model of diabetes. J. Biol. Chem..

[23] Abdul-Ghani M.A., DeFronzo R.A. (2008). Inhibition of renal glucose absorption: A novel strategy for achieving glucose control in type 2 diabetes mellitus. Endocr. Pract..

[24] Thorens B., Mueckler M. (2010). Glucose transporters in the 21st century. Am. J. Physiol. Endocrinol. Metab..

[25] Bays H. (2013). Sodium glucose co-transporter type 2 (SGLT2) inhibitors: Targeting the kidney to improve glycemic control in diabetes mellitus. Diabetes Ther..

[26] Oku A., Ueta K., Arakawa K., Ishihara T., Nawano M., Kuronuma Y., Matsumoto M., Saito A., Tsujihara K., Anai M. (1999). T-1095, an inhibitor of renal Na^+^-glucose cotransporters, may provide a novel approach to treating diabetes. Diabetes.

[27] Katsuno K., Fujimori Y., Takemura Y., Hiratochi M., Itoh F., Komatsu Y., Fujikura H., Isaji M. (2007). Sergliflozin, a novel selective inhibitor of low-affinity sodium glucose cotransporter (SGLT2), validates the critical role of SGLT2 in renal glucose reabsorption and modulates plasma glucose level. J. Pharmacol. Exp. Ther..

[28] Fujimori Y., Katsuno K., Nakashima I., Ishikawa-Takemura Y., Fujikura H., Isaji M. (2008). Remogliflozin etabonate, in a novel category of selective low-affinity sodium glucose cotransporter (SGLT2) inhibitors, exhibits antidiabetic efficacy in rodent models. J. Pharmacol. Exp. Ther..

[29] Bickel M., Brummerhop H., Frick W., Glombik H., Herling A.W., Heuer H.O., Plettenburg O., Theis S., Werner U., Kramer W. (2008). Effects of AVE2268, a substituted glycopyranoside, on urinary glucose excretion and blood glucose in mice and rats. Arzneimittelforschung.

[30] Derdau V., Fey T., Atzrodt J. (2010). Synthesis of isotopically labelled SGLT inhibitors and their metabolites. Tetrahedron.

[31] Fujimori Y., Katsuno K., Ojima K., Nakashima I., Nakano S., Ishikawa-Takemura Y., Kusama H., Isaji M. (2009). Sergliflozin etabonate, a selective SGLT2 inhibitor, improves glycemic control in streptozotocin-induced diabetic rats and Zucker fatty rats. Eur. J. Pharmacol..

[32] Katsuno K., Fujimori Y., Ishikawa-Takemura Y., Isaji M. (2009). Long-term treatment with sergliflozin etabonate improves disturbed glucose metabolism in KK-A(y) mice. Eur. J. Pharmacol..

[33] Hussey E.K., Clark R.V., Amin D.M., Kipnes M.S., O’Connor-Semmes R.L., O’Driscoll E.C., Leong J., Murray S.C., Dobbins R.L., Layko D. (2010). Single-dose pharmacokinetics and pharmacodynamics of sergliflozin etabonate, a novel inhibitor of glucose reabsorption, in healthy volunteers and patients with type 2 diabetes mellitus. J. Clin. Pharmacol..

[34] Hussey E.K., Dobbins R.L., Stoltz R.R., Stockman N.L., O’Connor-Semmes R.L., Kapur A., Murray S.C., Layko D., Nunez D.J. (2010). Multiple-dose pharmacokinetics and pharmacodynamics of sergliflozin etabonate, a novel inhibitor of glucose reabsorption, in healthy overweight and obese subjects: A randomized double-blind study. J. Clin. Pharmacol..

[35] Dobbins R.L., O’Connor-Semmes R., Kapur A., Kapitza C., Golor G., Mikoshiba I., Tao W., Hussey E.K. (2012). Remogliflozin etabonate, a selective inhibitor of the sodium-dependent transporter 2 reduces serum glucose in type 2 diabetes mellitus patients. Diabetes Obes. Metab..

[36] Mudaliar S., Armstrong D.A., Mavian A.A., O’Connor-Semmes R., Mydlow P.K., Ye J., Hussey E.K., Nunez D.J., Henry R.R., Dobbins R.L. (2012). Remogliflozin etabonate, a selective inhibitor of the sodium-glucose transporter 2, improves serum glucose profiles in type 1 diabetes. Diabetes Care.

[37] Hussey E.K., Kapur A., O’Connor-Semmes R., Tao W., Rafferty B., Polli J.W., James C.D., Dobbins R.L. (2013). Safety, pharmacokinetics and pharmacodynamics of remogliflozin etabonate, a novel SGLT2 inhibitor, and metformin when co-administered in subjects with type 2 diabetes mellitus. BMC Pharmacol. Toxicol..

[38] Kapur A., O’Connor-Semmes R., Hussey E.K., Dobbins R.L., Tao W., Hompesch M., Smith G.A., Polli J.W., James C.D., Mikoshiba I. (2013). First human dose-escalation study with remogliflozin etabonate, a selective inhibitor of the sodium-glucose transporter 2 (SGLT2), in healthy subjects and in subjects with type 2 diabetes mellitus. BMC Pharmacol. Toxicol..

[39] Sykes A.P., O’Connor-Semmes R., Dobbins R., Dorey D.J., Lorimer J.D., Walker S., Wilkison W.O., Kler L. (2015). Randomized trial showing efficacy and safety of twice-daily remogliflozin etabonate for the treatment of type 2 diabetes. Diabetes Obes. Metab..

[40] Sykes A.P., Kemp G.L., Dobbins R., O’Connor-Semmes R., Almond S.R., Wilkison W.O., Walker S., Kler L. (2015). Randomized efficacy and safety trial of once-daily remogliflozin etabonate for the treatment of type 2 diabetes. Diabetes Obes. Metab..

[41] O’Connor-Semmes R., Walker S., Kapur A., Hussey E.K., Ye J., Wang-Smith L., Tao W., Dobbins R.L., Cheatham B., Wilkison W.O. (2015). Pharmacokinetics and pharmacodynamics of the SGLT2 inhibitor remogliflozin etabonate in subjects with mild and moderate renal impairment. Drug Metab. Dispos..

[42] Link J.T., Sorensen B.K. (2000). A method for preparing *C*-glycosides related to phlorizin. Tetrahedron Lett..

[43] Meng W., Ellsworth B.A., Nirschl A.A., McCann P.J., Patel M., Girotra R.N., Wu G., Sher P.M., Morrison E.P., Biller S.A. (2008). Discovery of dapagliflozin: A potent, selective renal sodium-dependent glucose cotransporter 2 (SGLT2) inhibitor for the treatment of type 2 diabetes. J. Med. Chem..

[44] Han S., Hagan D.L., Taylor J.R., Xin L., Meng W., Biller S.A., Wetterau J.R., Washburn W.N., Whaley J.M. (2008). Dapagliflozin, a selective SGLT2 inhibitor, improves glucose homeostasis in normal and diabetic rats. Diabetes.

[45] Komoroski B., Vachharajani N., Feng Y., Li L., Kornhauser D., Pfister M. (2009). Dapagliflozin, a novel, selective SGLT2 inhibitor, improved glycemic control over 2 weeks in patients with type 2 diabetes mellitus. Clin. Pharmacol. Ther..

[46] List J.F., Woo V., Morales E., Tang W., Fiedorek F.T. (2009). Sodium-glucose cotransport inhibition with dapagliflozin in type 2 diabetes. Diabetes Care.

[47] Wilding J.P., Norwood P., T’joen C., Bastien A., List J.F., Fiedorek F.T. (2009). A study of dapagliflozin in patients with type 2 diabetes receiving high doses of insulin plus insulin sensitizers: Applicability of a novel insulin-independent treatment. Diabetes Care.

[48] Ferrannini E., Ramos S.J., Salsali A., Tang W., List J.F. (2010). Dapagliflozin monotherapy in type 2 diabetic patients with inadequate glycemic control by diet and exercise: A randomized, double-blind, placebo-controlled, phase III trial. Diabetes Care.

[49] Bailey C.J., Gross J.L., Pieters A., Bastien A., List J.F. (2010). Effect of dapagliflozin in patients with type 2 diabetes who have inadequate glycaemic control with metformin: A randomised, double-blind, placebo-controlled trial. Lancet.

[50] Strojek K., Yoon K.H., Hruba V., Elze M., Langkilde A.M., Parikh S. (2011). Effect of dapagliflozin in patients with type 2 diabetes who have inadequate glycaemic control with glimepiride: A randomized, 24-week, double-blind, placebo-controlled trial. Diabetes Obes. Metab..

[51] Nauck M.A., Del Prato S., Meier J.J., Durán-García S., Rohwedder K., Elze M., Parikh S.J. (2011). Dapagliflozin versus glipizide as add-on therapy in patients with type 2 diabetes who have inadequate glycemic control with metformin: A randomized, 52-week, double-blind, active controlled noninferiority trial. Diabetes Care.

[52] Rosenstock J., Vico M., Wei L., Salsali A., List J.F. (2012). Effects of dapagliflozin, an SGLT2 inhibitor, on HbA1c, body weight, and hypoglycemia risk in patients with type 2 diabetes inadequately controlled on pioglitazone monotherapy. Diabetes Care.

[53] Nomura S., Sasamaki S., Hongu M., Kawanishi E., Koga Y., Sakamoto T., Yamamoto Y., Ueta K., Kimata H., Nakayama K. (2010). Discovery of canagliflozin, a novel *C*-glucoside with thiophene ring, as sodium-dependent glucose cotransporter 2 inhibitor for the treatment of type 2 diabetes mellitus. J. Med. Chem..

[54] Rosenstock J., Aggarwal N., Polidori D., Zhao Y., Arbit D., Usiskin K., Capuano G., Canovatchel W., Canagliflozin DIA 2001 Study Group (2012). Dose-ranging effects of canagliflozin, a sodium-glucose cotransporter 2 inhibitor, as add-on to metformin in subjects with type 2 diabetes. Diabetes Care.

[55] Devineni D., Morrow L., Hompesch M., Skee D., Vandebosch A., Murphy J., Ways K., Schwartz S. (2012). Canagliflozin improves glycaemic control over 28 days in subjects with type 2 diabetes not optimally controlled on insulin. Diabetes Obes. Metab..

[56] Stenlöf K., Cefalu W.T., Kim K.A., Alba M., Usiskin K., Tong C., Canovatchel W., Meininger G. (2013). Efficacy and safety of canagliflozin monotherapy in subjects with type 2 diabetes mellitus inadequately controlled with diet and exercise. Diabetes Obes. Metab..

[57] Yale J.F., Bakris G., Cariou B., Yue D., David-Neto E., Xi L., Figueroa K., Wajs E., Usiskin K., Meininger G. (2013). Efficacy and safety of canagliflozin in subjects with type 2 diabetes and chronic kidney disease. Diabetes Obes. Metab..

[58] Grampler R., Thomas L., Eckhardt M., Himmelsbach F., Sauer A., Sharp D.E., Bakker R.A., Mark M., Klein T., Eickelmann P. (2012). Empagliflozin, a novel selective sodium glucose cotransporter-2 (SGLT-2) inhibitor: Characterisation and comparison with other SGLT-2 inhibitors. Diabetes Obes. Metab..

[59] Mudaliar S., Polidori D., Zambrowicz B., Henry R.R. (2015). Sodium-glucose cotransporter inhibitors: Effects on renal and intestinal glucose transport: From bench to bedside. Diabetes Care.

[60] Sato S., Takeo J., Aoyama C., Kawahara H. (2007). Na^+^-glucose cotransporter (SGLT) inhibitory flavonoids from the roots of *Sophora flavescens*. Bioorg. Med. Chem..

[61] Yang J., Yang X., Wang C., Lin Q., Mei Z., Zhao P. (2015). Sodium-glucose-linked transporter 2 inhibitors from *Sophora flavescens*. Med. Chem. Res..

[62] Morita H., Deguchi J., Motegi Y., Sato S., Aoyama C., Takeo J., Shiro M., Hirasawa Y. (2010). Cyclic diarylheptanoids as Na^+^-glucose cotransporter (SGLT) inhibitors from *Acer nikoense*. Bioorg. Med. Chem. Letter..

[63] Arai H., Hirasawa Y., Rahman A., Kusumawati I., Zaini N.C., Sato S., Aoyama C., Takeo J., Morita H. (2010). Alstiphyllanines E-H, picraline and ajmaline-type alkaloids from *Alstonia macrophylla* inhibiting sodium glucose cotransporter. Bioorg. Med. Chem..

[64] Shimokawa Y., Akao Y., Hirasawa Y., Awang K., Hadi A.H., Sato S., Aoyama C., Takeo J., Shiro M., Morita H. (2010). Gneyulins A and B, stilbene trimers, and noidesols A and B, dihydroflavonol-*C*-glucosides, from the bark of *Gnetum gnemonoides*. J. Nat. Prod..

[65] He X., Fang J., Huang L., Wang J., Huang X. (2015). *Sophora flavescens* Ait.: Traditional usage, phytochemistry and pharmacology of an important traditional Chinese medicine. J. Ethnopharmacol..

[66] Nagai M., Kubo M., Fujita M., Inoue T., Matsuo M. (1978). Studies on the constituents of Aceraceae plants. II. Structure of aceroside I, a glucose of a novel cyclic diarylheptanoids from *Acer nikoense* Maxim. Chem. Pharm. Bull. (Tokyo).

[67] Changwichit K., Khorana N., Suwanborirux K., Waranuch N., Limpeanchob N., Wisuitiprot W., Suphrom N., Ingkaninan K. (2011). Bisindole alkaloids and secoiridoids from *Alstonia macrophylla* Wall. ex G. Don. Fitoterapia.

[68] Khyade M.S., Kasote D.M., Vaikos N.P. (2014). *Alstonia scholaris* (L.) R. Br. and *Alstonia macrophylla* Wall. ex G. Don: A comparative review on traditional uses, phytochemistry and pharmacology. J. Ethnopharmacol..

[69] Huang T., Shen P., Shen Y. (2005). Preparative separation and purification of deoxyschisandrin and gamma-schisandrin from *Schisandra chinensis* (Turcz.) Baill by high-seed counter-current chromatography. J. Chromatogr. A.

[70] Chan S.W. (2012). Panax ginseng, Rhodiola rosea and Schisandra chinensis. Int. J. Food Sci. Nutr..

[71] Qu Y., Chan J.Y., Wong C.W., Cheng L., Xu C., Leung A.W., Lau C.B. (2015). Antidiabetic effect of Schisandrae Chinensis Fructus involves inhibition of the sodium glucose cotransporter. Drug Dev. Res..

[72] INVOKANA^®^ (Canagliflozin) Prescribing Information. http://www.accessdata.fda.gov/drugsatfda_docs/label/2016/204042s015s019lbl.pdf.

[73] JARDIANCE^®^ (Empagliflozin) Prescribing Information. http://www.accessdata.fda.gov/drugsatfda_docs/label/2016/204629s005lbl.pdf.

[74] Wu J.S., Peng Y.H., Wu J.M., Hsieh C.J., Wu S.H., Coumar M.S., Song J.S., Lee J.C., Tsai C.H., Chen C.T. (2010). Discovery of non-glycoside sodium-dependent glucose co-transporter 2 (SGLT2) inhibitors by ligand-based virtual screening. J. Med. Chem..

[75] Devineni D., Vaccaro N., Murphy J., Curtin C., Mamidi R.N., Weiner S., Wang S.S., Ariyawansa J., Stieltjes H., Wajs E.P. (2015). Effects of rifampin, cyclosporine A, and probenecid on the pharmacokinetic profile of canagliflozin, a sodium glucose co-transporter 2 inhibitor, in healthy participants. Int. J. Clin. Pharmacol. Ther..

